# Parental Burnout: When Exhausted Mothers Open Up

**DOI:** 10.3389/fpsyg.2018.01021

**Published:** 2018-06-26

**Authors:** Sarah Hubert, Isabelle Aujoulat

**Affiliations:** ^1^Psychological Sciences Research Institute, Université catholique de Louvain, Louvain-la-Neuve, Belgium; ^2^Institute of Health and Society, Université catholique de Louvain, Brussels, Belgium

**Keywords:** exhaustion, lived experience, parental burnout, mother–child relationship, phenomenological interpretative analysis, qualitative methods

## Abstract

Until recently, research conducted on parental exhaustion was exclusively concerned with parents of sick children. However, situations where exhaustion occurs as a result of being physically and emotionally overwhelmed by one’s parental role in the absence of a child’s condition is gaining increasing interest. The aim of our study was to give voice to exhausted mothers, in order to get a better understanding of what it means to be exhausted in relation with one’s parental role, from the perspective of those who have experienced it. We referred to phenomenological interpretative analysis for methods of data collection and data analysis, and included five mothers who were each interviewed twice. Our analysis revealed a superordinate theme of fear, which was central in every aspect of the mothers’ accounts of their experiences, from the fear to not be a good enough mother to the fear related to unlearning control and experiencing discontinuity of one’s sense of self. Our results call for the development of specific interventions to prevent, anticipate, or treat the phenomenon of exhaustion in parents, so as to help them and their children cope better with these situations of extreme vulnerability, which are often reinforced by senses of guilt, shame, and loneliness.

## Introduction

The phenomenon of *burnout* refers to a specific syndrome of exhaustion related to prolonged situations of emotional imbalance, where the burden of perceived stress exceeds personal resources to cope with it. While the phenomenon of *professional* burnout, which was described by Freudenberger (1974) to define a syndrome of exhaustion related to working conditions, is nowadays well known (Maslach et al., 2001), that of *parental* burnout, i.e., situations where exhaustion occurs as a result of being physically and emotionally overwhelmed by one’s parental role, still deserves attention. The concept had been tentatively put forward in the 1980s in the United States (Procaccini and Kiefaver, 1983), before being put forward again only 4 years ago, in Europe (Holstein, 2014).

Until recently, research conducted on parental exhaustion was exclusively concerned with parents of sick children (e.g., Lindhal Norberg, 2007; Lindström et al., 2010; Karadavut and Uneri, 2011; Lindahl Norberg et al., 2014). Our study is part of a broader research project initiated in 2015 in Belgium, with the aim to better understand, define, diagnose, and prevent or treat parental burnout in families with healthy children (Roskam and Mikolajczak, 2015). The first results of this ongoing research suggest that parental burnout may potentially affect up to 14% of parents (Raes, 2018). Parental burnout is characterized by three aspects that echo the characteristics of professional burnout while differentiating from it by being in relation with parenting: (i) Physical and emotional exhaustion; (ii) emotional distancing from one’s children, and (iii) a sense of incompetency in one’s parenting role (Mikolajczak, 2018). Moreover, based on the identification of specific risk factors of parental burnout (Le Vigouroux et al., 2017; Mikolajczak et al., 2017), a first measure of parental exhaustion, the *Parental Burnout Inventory* scale, has recently been created and validated (Roskam et al., 2017).

Although mothers with a parental burnout syndrome appear to share some of the characteristics of maternal post-partum depression (Eg. Fatigue, lack of energy), maternal burnout differs in that (i) it occurs in mothers with children over 18 months of age; (ii) it is predominantly linked to parental traits and to a lesser extent to social and marital factors; (iii) the depressive mood is not generalized but experienced in relation to one’s parenting role and tasks (Nunes Tuna, 2018).

To the best of our knowledge, the perspective of exhausted mothers regarding their own experience of parental burnout has never been explored. The aim of our study was therefore to give voice to exhausted mothers, in order to get a better understanding of what it means to be exhausted in relation with one’s parental role, from the perspective of those who have experienced it.

## Materials and Methods

We aimed to understand better the lived experience of parental burnout, from the perspective of the mothers who have experienced it. We referred to *interpretative phenomenological analysis* (IPA) (Smith et al., 1999, 2009) for data collection and analysis. This choice is congruent with our aim to uncover the meaning given by these mothers to their singular experiences.

### Participants

Although parental burnout is not limited to mothers but also occurs in fathers, for the sake of homogeneity in our sample, only mothers were included in this study. Moreover, in order to avoid possible confusion with postpartum depression, we only included mothers with children over 18 months. Last but not least, in order to be included in our study, the mothers needed to be well aware of their experience of exhaustion in relation to their parenting role, and willing to share their experiences with us. We therefore approached our participants through an internet forum and a facebook account dedicated to maternal exhaustion^1,2^ as the participants in these discussions would have already self-diagnosed themselves as being exhausted and suffering from burnout symptoms in relation to their parenting role. Following a short announcement posted on the internet, 10 women volunteered to participate, of which 5 were excluded for one of the following reasons: being a mother of a child under 18 months of age; having experienced a job burnout before the parental burnout; and reporting generalized depressive symptoms. After about 6 weeks, the recruitment was closed.

Congruently with the principles of IPA, where the importance and relevance of the findings does not depend on the size of the sample but on the richness of the narratives provided by a few purposively chosen cases on the one hand, and the researcher’s skill to meaningfully interact with the participants and the data during the analytical process (Brocki and Wearden, 2006; Smith, 2011), our sample was deliberatively kept small (Reid et al., 2005), thus allowing for homogeneity and an in-depth exploration of feelings and meanings associated with parental exhaustion.

The five (5) women who participated in our study were aged 30 to 42 (mean 38.6 years) and happened to all have two children, the youngest child being two, and the eldest 14 (mean 7.2 years). Four of them were living in partnership or married with their children’s father at the time of the interview, and one was divorced. As far as their employment status is concerned, two participants were working full time, and another one was working half-time. Moreover, one participant had deliberately chosen to stop working, while another one was on sick leave at the time of the interview.

### Data Collection

All the interviews were conducted by the first author over an 8-month period (from 25/02/16 to 06/10/16) in 5 different cities in Belgium. The data was collected through in depth-interviews using a flexible interview guide, starting with a very broad question “*Tell me about your experience of getting exhausted as a mother*,” and was further allowed to evolve as a natural discussion driven by the participant, with the researcher building in prompts and questions around several broadly predefined topics in relation with the research objective, i.e., events, feelings, and meanings associated with being exhausted.

Every woman was interviewed twice: a first exploratory interview was followed 4–6 weeks later by a second interview, after the transcript of the first interview had been sent to the participant. The second interview was meant to allow for the participant to go more in-depth or amend her narrative where necessary, thus allowing for the emergence of a co-constructed meaning rather than the interpretation relying on the researchers alone. Mean durations were 2 hours 24 min (2 h 15 min–2 h 45 min) for the first interview, and 1 hour 26 min (1 h 15 min–2 h) for the second interview. According to the participants’ preferences, the interviews took place either at their house (*n* = 4) or at the researcher’s house (*n* = 1). The participants were met on their own, except for one situation where the husband and child were present in the house but did not interfere with the interview process. An informed consent form was signed before every interview.

### Data Analysis

Every interview was audiotaped with the consent of the participant, and transcribed at verbatim. The transcripts were analyzed inductively by the first author, who is a clinical psychologist, and discussed for credibility checks with the second author, who has extensive experience in qualitative analysis in relation with lived experience of chronic conditions. Congruently with principles of interpretative phenomenological analysis (IPA), the discussion with a second researcher (co-author) was meant to enrich the understanding of the meaning derived from the transcripts, not to reach consensus (Brocki and Wearden, 2006). Following multiple readings, meaningful descriptive and conceptual themes were created, first in relation to every single narrative and then across the narratives, by looking at connections between the themes.

Our analysis revealed a superordinate theme of fear, which was central in every aspect of the mothers’ accounts of their experience of becoming and being exhausted. Our results are organized according to this central theme, from the fear to not be a good enough mother to the fear related to unlearning control and experiencing discontinuity of one’s sense of self. Our results are illustrated with relevant citations from the participants, whose names have been changed.

## Results

### From Overinvestment to Exhaustion: The Fear of Not Being a Good Enough Mother

All the participants reported that they had overinvested their maternal role over a long period of time before becoming exhausted. They described themselves as being in charge of everything at home, without ever asking for help: *”Always being here and there, doing several things at the same time (…). We are the one who knows, so we keep spending our time and energy… we keep preparing meals, supervising homework, bathing, cleaning, etc.” (Mei).* At the time, they were not aware of their doing too much: “*One does not realize that one is going too far and should stop or change [...] On the contrary, I used to feel that I was not doing enough. That’s crazy…*” *(Bénédicte).* Another participant conveyed how rewarding it used to be to feel that her role was unique, and that she was able to meet all of her children’s needs: “*I liked it, there was only me to do it; it was nice to be the super-mother.”*

Underlying the participants’ overinvestment of their parental role, which led them to anticipate and react to all of their children’s wishes, were a desire for perfection, a sense of self-pressure and a tendency to anticipate their children’s future.

#### Perfectionism

All the participants conveyed that they had a great sense of duty, were often in doubt about their own abilities, and were very self-demanding. “*No doubt I am a hyper perfectionist, I would see myself as the perfect mother who would never be angry, who would carefully listen to her children, in an always benevolent way.” (Elisabeth).* Good was not enough for these mothers, who would always strive to do better: “*We are driven by a kind of archetype of a perfect behavior which leads us to set goals that are hard to achieve. When we do not achieve our goals, we get a sense of failure. Maybe such goals are irrealistic. [...] I think that’s why I got exhausted. If I had been able to let go instead of wanting everything to be perfect, maybe I would not have gone crazy” (Violette).* For others, perfectionism was not necessarily defined in relation to the desire to achieve specific goals, but was associated with a continuous sense of doubt and uncertainty regarding their very ability to be a mother “*I did not even believe that I could do better; I used to always question my very ability to get things right. I used to be always in doubt about myself, and therefore I would continue to seek to do more and better… all the time.*” *(Bénédicte).*

#### Self-Pressure

The participants’ desire to be perfect was consciously or unconsciously associated with a strong sense of self-pressure, both in relation with internalized social norms of positive parenting, or with self-constructed norms. “*They [the children] must be clean, well dressed, well behaved... They need to be actively entertained*, should *not be put in front of the TV... You get all these kinds of clichés, you know, which you try to comply with (Violette).* The internalized social norms were expressed also in relation to feeding: “*No ready-to-serve baby food… this is no good… therefore I would prepare fresh vegetables every day. Even in days where we would go to see Grand-pa and Grand-ma, for instance. I would get up one hour early so that I’d be able to put the dinner and snack in the Babycook. Crazy thing!*” *(Bénédicte).* The participants described their sense of self-pressure as very strong, in relation with sometimes inherited norms and values which they would not derogate to, even when advised to by others: “*They told me once at the nursery: ‘Listen, you are here as early as 7 am, we can feed your children here at the nursery’. And me: ‘No, no, no, they should eat at home with us’*… *for the sake of respecting the principle of having family meals [...] As a matter of fact, I remember these morning meals as hell ‘Hurry up, go, drink your milk. Quick! Quick!’ (Véronique).* The participants conveyed their urge to transmit values with the aim to secure a strong emotional and social basis in their children: “*this drives me to spend my energy like crazy to make my children feel valued and strengthened in terms of their emotional security and so on... [...] It had become sort of obsessional.” (Mei ).* Still the doubt was there, while struggling to find the right balance: “*(…) I was afraid of giving my child the impression that I did not care enough for him and yet, at the same time, afraid of taking too much care of him [...] One should give them all the affection they need, but not too much so that they can be autonomous, not depend on their mother’s affection... so that if I was going to die the next day, this would not be the end of the world for him. Struggling to find the right balance… thinking back to it, I think that this is what got me exhausted in the end.” (Bénédicte).*

In addition to wanting to comply with expectations in relation to well identified social norms, the participants also reported that they had lacked internal norms to give them an inner sense of certainty and security, thus adding to their sense of pressure in an insidious way: “*I think that this got me exhausted too… the fact that I never actually knew what exactly to expect from me as a mother... I was always in doubt, and never settled my mind...” (Bénédicte).* While questioning their identity as a mother, some participants recalled their relationship with their own mothers which may have played an important role in how they saw themselves as mothers. For some of them, an idealized image of their mother, might have added to their sense of pressure, by raising high expectations to live up to: “*There is probably the fact that it seemed normal to exhaust myself for my children, because my mother had exhausted herself for us.” (Bénédicte).* By contrast, another participant recalled that her needs as a child had not been met by her own mother: “*I clearly made my children my priority. It has been really painful for me to have a bad relationship with my mother [...] Yes, for sure I defined my own way of being a mother, against that of my mother. Contrary to what she used to be like, I had a desire to be all and everywhere for my own children… I wanted to be able to give them everything they needed.”* (Elisabeth)

#### Projection

In addition to wanting to fulfill all of their children’s needs all the time, the participants in our sample were also over concerned with their children’s future, and their own responsibility in preparing for their children’s future: “*I am the one who is responsible for what they will be later… what they will become depends on what I do now…”* (Bénédicte). Their main goal as mothers was to pave the way for future adults who should be fulfilled as human beings, self-reliant, and independent: “*As children, they absorb everything that is told or done. Depending on their character and the life events they meet, what they get may be later turned into positive experiences or trauma… you never know… this is what kills me: the awareness that everything I do contributes to his personality. So what if I am wrong, if I do bad?” (Violette).* The mothers’ concern for their responsibility in preparing for their children’s future may become overwhelming: “*Well… What I experience, is fear actually. The fear of who my child is going to be, the fear that my child believes he is not loved enough, the fear that... [...] I have this on my mind all the time… to know that what I do now is what will contribute to make my children happy or unhappy in the future. My only goal is to make them happy later. That’s my problem: the fact that my goal is not to make them happy now, but to make them happy later. This used to be a big, big problem.” (Bénédicte)* Because of this overwhelming concern for the future of their children, the mothers were not able to enjoy the present, which added to their sense of pressure.

### From Tiredness to Automatic Behavior and Emotional Distancing: Fear Related to Unlearning Control

We described in the previous paragraph the slippery road which may slowly drive the mothers from being actively and deliberately overinvested in the care to their children, to painfully questioning their competence and perceiving the demands put on them as exceeding their resources and possibilities. The participants recalled their experiences highlighting the progressive feelings of frustration, irritation, and saturation that had paved the way for their actual breakdown.

#### Intense Physical and Emotional Fatigue

All the participants reported to have experienced very intense fatigue, closely interconnected with perceived stress and anxiety: “*I kept thinking over and over again… A the same time I was filled up with anxiety [...] I think that too got me down… when you do not get enough sleep, for sure you cannot physically cope : you lose your patience, you get exhausted and angry. This in turn has an impact on your mental health as your reasoning abilities are thwarted.” (Violette).* At this point of intense physical and emotional fatigue, it feels like the body says “stop” and pervading agitation, tension and nervousness are experienced: “*If I had to describe it, I would say that it’s like suffering a giant fatigue which goes along with a kind of constant nervousness. Sometimes I feel like all my nerves are agitated, as if everything were upside down inside me [...] as if it was all foggy inside my head. That’s the kind of fatigue it’s like” (Elisabeth).* Specific physical symptoms were also individually reported: “*(…) pain in the neck, tinnitus, headaches... [...] I was also dizzy, and felt sick as if I had flu, everything was like turning around me and making me dizzy*” *(Véronique).* As they felt exhausted, the participants reported to have had no access to their inner strength anymore: “*At this point, it’s too late, one has burned out all one’s resources and energy. It’s all empty inside. Just empty. Mentally and physically empty!” (Violette).*

#### Surviving as an Automatic Pilot

The above described state of intense fatigue, stress, and anxiety inevitably had an impact on the management of everyday life, including interactions with the children. At this stage, the exhausted mothers tended to develop an aversion toward any house related responsibilities and chores that had once been so important to them: “*I cannot stand these logistical responsibilities anymore. Indeed, this is one of the most difficult things to cope with in the aftermath: the fact that I have now developed a kind of allergy to responsibilities. I had enough of them, I cannot stand them anymore... even stupid little things such as organizing my daughters’ activities during the holidays… it was even physically difficult. Having to choose the type of activities they would do, thinking of how I would drive them there (…) all this had become too much for me” (Violette).*

Planning, anticipating, and doing projects became complicated. The participants who had hitherto as mothers engaged body and soul in the bringing up of their children with an idealized future prospect for them, slowly saw themselves become unable to think further than here and now: “*It was awful, because I used to wake up with zero energy and the smallest thing was really difficult. Just to think about what we would eat at night was making me anxious” (Elisabeth).* The participants illustrated how they would feel overwhelmed by any medium or long term project: “*I was unable to make any kind of project, I was just there… [...] At this point there is no way you can take some distance and look at the future. (…) What makes it so complicated, is that it is impossible to think further than here and now. The thought that it would be over one day because she would eventually grow up never occurred to me. There was no future to imagine (…) To think ahead was causing too much anxiety, so it was better for me not to” (Violette).*

The participants conveyed to have been exclusively focused on the uncomfortable present time, which they had not enjoyed. They recalled struggling to survive in an automatic mode, as if they had been robots: “*Yes, the least thing was requesting so much energy... I did not have that energy anymore…I was just trying to survive… I was like a robot” (Elisabeth).* The smallest change or unforeseen event would be perceived as violent; there was no room for improvisation and creativity. “*I used to experience the least change as a catastrophe. To change the schedule was unimaginable... [...] It would create too much chaos. There was a kind of structure in my chaos, and this was not to be moved, otherwise it would put me down” (Veronique).* The mothers at this stage of exhaustion saw themselves as locked into blinders, both protective and threatening: “*It was not possible to stop the routine, nor to learn to change… I would not allow myself to learn other ways of doing things, to change any habit that was not good. I preferred to keep running fast and sticking to my habits rather than to stop and think. I was like a robot actually, doing things automatically, with no energy too think further.” (Véronique).*

#### Unbonding From Their Children and Losing Control

As they got exhausted, the participants would develop mechanical gestures in their interactions with their children: “*It is important to spend enough time with her. So I try. But it doesn’t come naturally.” (Violette)*. At this stage, the relationship to their children was described as something burdensome instead of rewarding: “*I was a mess at home, I was unable to cope. Everything was chaotic. I did not enjoy being with my children. (…) I would just shout all the time. I was not sharing anything with them, just controlling all the time (…) I never sat down with my children to share a pleasant moment with them” (Véronique).* To interact with their children, play with them, or simply be with them may become so burdensome that a mental and emotional separation occurs: “*I was there physically but I was mentally absent”* (Violette). This could lead to ignoring one’s children: “*I was not aware of my children anymore. I think I did not want to admit it to myself*” (Bénédicte).

At some point, the very presence of their children would be perceived as unbearable and lead to uncontrollable impulsive reactions: “*I kept losing control with my first daughter. I could not stand her anymore, actually [...] I would all of a sudden burst out screaming hysterically, with a huge violence. A huge verbal violence [...] It was like an impulse I was totally unable to control. I would have no control at all. [...] The violent outburst would originate in my guts like a storm and I would know no way to stop it [...] One can become very violent just with words. As a matter of facts, I used to sometimes say horrible things to my daughter, stuff like: ‘I’m so tired of you, I’m sick of the child you are.” It’s awful for a child to hear that...” (Elizabeth).* Violence may be expressed not only in words but in gestures as well, when everything goes completely out of control, as one of the participants recalled*:* “*I was tired and wanting to go to bed. So I became irritated and started shouting. ‘Get dressed, get dressed, get dressed!’ I kept shouting stronger and stronger, getting more and more angry [...] and then, as I was in her room, I cried and I knocked the wardrobe hard with my fist [...] So I knocked the wardrobe and it hurt. It often hurts when I get so angry but apparently it never hurts enough to stop me. Yet, that time, I made a hole in the furniture. And there, my daughter, as small as she was (only 80 cm high) looked at me and said, ‘Mom, you made a hole.’ That’s what I call ‘my crisis’, that’s when I started realizing…” (Bénédicte).*

Like Bénédicte, the mothers who participated in our study were usually able to redirect their aggressiveness toward objects rather than their children. Extremely violent thoughts and behaviors were yet frequent : “*There have been times... I remember one particularly difficult moment when she did not want to take a nap. And me, I wanted her to take that nap, because it was just not possible for me to further manage the afternoon if she was not going to sleep. And, there as I had just dragged her in bed, in such a way that her head had struck the edge of the bed, I suddenly felt an urge inside me to box her to death. It was a horrible thought; it was horrible to have this kind of impulse in me.” (Elisabeth).* Such events were extremely painful for the mothers who experienced them. We saw earlier that exhausted mothers would experience distance and even withdrawal from their children’s emotions. In periods of intense crisis, such withdrawal might be experienced as a desire to flee, either for a period of time, or more radically: “*I happened to think that I wanted to kill myself. This would have been a way to escape, to not have to make decisions anymore, to not have to manage anything anymore. At that time, I felt I should either kill myself, or leave the girls. It was one or the other. There have been really times like that in that period. I used to think that there was no other way out. I was so much trapped in it, that I was not able to take the smallest step back. At the highest of the crisis, everything appears as disproportionately difficult.” (Violette).* The idea to commit suicide is not an unusual thought for exhausted mothers, although it has fortunately not been put into practice by none of the participants in our study.: “*well, If I feel I cannot cope with it anymore… well I am not sure if I could do it or not… but I have already considered… well, I have sometimes thought that changing the direction of my car may be quickly done. I am aware that I need to be beware of myself...” ( Mei ).*

### Maternal Exhaustion as Major Suffering: Fear Related to Experiencing Discontinuity of One’s Sense of Self

The previously discussed elements shed some light on the nature and extent of the distress experienced by the participants. Although their personality probably did not fundamentally change, all reported feeling different and strange: “*I felt for several months as if I was disconnected from myself. It’s hard to understand for someone who has not experienced it, but I felt as if I was living besides myself. (…) even when I looked at me into the mirror, I could not see myself” (Elisabeth).* Not only did the mothers feel strange and disconnected as they got exhausted, they also reported to have acted strangely and inappropriately, sometimes with violence, under the influence of an uncontrollable impulse. The participants recalled such experience with self-hate and fear of themselves. Moreover, they reported three inter-related predominant feelings in relation to their experiences, that of shame, loneliness and guilt.

#### Self-Hate and Fear

Their inability to control themselves and tendency to react violently in certain situations was among the mothers’ most traumatic experiences as they would never have suspected such hidden side of their personality: “*It was so scary to face the darkest parts that were hidden within myself, and which I had no idea of. Yes, I was terrified that I had that kind of thoughts and instincts towards those most precious to me in the world. It was terrifying, I was so afraid of myself” (Elizabeth).* Another participant: “*I did not recognize myself. Even now I do not recognize myself… in the sense that I would never have imagined to be like that, capable of such strong mood swings and also violent with my own children. In that sense, I no longer recognize myself.*” (Violette)

The experience of negative feelings toward their children and the loss of self-control undermined the mothers’ self-esteem and sense of competence: “*As I faced these difficulties with my children, and saw the parts most horrible of me, I got so scared and destabilized that I completely stopped feeling confident as a mother [...] Family life had become like hell, and I was unable to manage… quite the opposite of what I had intended” (Elisabeth).* As the gap between their aspirations and reality widens, the exhausted mothers come to endorse a sense of failure that goes as far as self-hate: *“At times of crisis like that, I hate myself for not knowing how to manage.” (Mei).* From looking critically at what they *do*, the exhausted mothers’ self-depreciation moves to looking critically at what they *are* intrinsically. *“For me, the biggest difficulty with that burnout was to look at who I had become… a vegetable, a person without spirit, without anything... I was so negative about myself and everything that had to do with me… I could not even look at myself in the mirror, it was impossible” (Véronique).* The participants reported feeling trapped in horrible feelings, not knowing how to escape although they had so much wanted to. *“At that point, there is no way out that you could think of… except maybe… well, a drastic issue [...] It was safer to stop thinking ahead, because this may have otherwise led to… divorcing… or abandoning the girls… or worse” (Violette).*

#### Shame

The participants reported feeling that they did not deserve to be a mother. This was a distressing feeling which they could, however, not share with others: *“Although I was very close to my mother, this was something I could not talk about. To think that myself and my life were a failure made me feel too uncomfortable.… I could not talk about it” (Véronique).* As already discussed, the normative idea of what defines a good mother was a source of pressure, adding to the sense of shame experienced by the participants: *“What makes me feel bad compared to others, is all that happens at home. I cannot speak about it. How could I explain to my colleagues that I am not even able to get up in the morning, in order to get them dressed, or that I haven’t bathed them for three days because I didn’t feel like it. I cannot speak that out, of course... Because I am so ashamed of it of course [...] That’s why I don’t say anything. Even if it has become quite common to hear people say they are upset by their children, it is much different to hear someone say “I do not like to take care of my children”. This is not tolerated, nor socially and culturally accepted” (Violette)*.

#### Loneliness

Along with the sense of shame and sometimes as a consequence of it, the participants reported a great sense of loneliness. As illustrated above, asking for help was not easy, thus adding to the burden that one is alone to cope with all kinds of expectations and responsibilities. The sense of loneliness was particularly acute when faced with situations where they were on their own with their children: *“The days spent with her were very unrewarding because I was just doing house chores, preparing food... [...] If you add the times for her naps, you suddenly end up completely cloistered at home [...] This meant feeling lonely, lacking social contacts… and as a consequence, the slippery slope directly down into hell...” (Elisabeth).* Beyond being factual, the mothers’ isolation may also be understood in a symbolic way. The perceived lack of support, help, recognition may induce a feeling of being abandoned and ignored, making any request for help extremely complicated: *“I could not share this. (…) I was very, very afraid of other people’s judgment and misunderstanding [...] moreover, there was a fear to be stigmatized. (…) People are very understanding for physical conditions, but are much less for any mental health related disorder.” (Elisabeth).*

The same participant highlighted how secret the experienced situations of loss of control may be kept, as they happen at home and nobody is aware of what happens: *“I think that our husbands do not realize what’s going on because they’re not present when such violence occurs. (…) such events occur only when one is alone with her children. That’s why I think husbands do not realize how serious it is “(Elisabeth).* Even if they had wanted to, the participants would find it extremely hard to express their feelings: “*I could never have told him what was going on because I simply did not have the words for it. So I preferred to keep it to myself, because I could not put my experience into words.” (Véronique).* Not only were the words lacking, but sometimes the very ability to think about such indescribable experience, was impeded by the state of exhaustion: *“I think he (husband) saw it from the beginning, but he did not know how to help me... As I was not able to speak about it, he did not know how to help. He asked me: ‘What can I do?’ I did not even know what to answer. I couldn’t have told him*, ‘*Take the kids and leave me alone’, could I?”* (*Violette*)

At this stage, the participants tended to keep thinking that they would fail to get things right, rather than being aware that they *were* not right, making it impossible for them to ask for help. *“Never did it occur to me that I was not well. Never! There was something wrong with me, but I never thought that I should have gone to a psychologist to talk about it. [...]as long as I was not told by someone, I couldn’t rely on myself to realize that I was not right. I kept blaming myself for doing things wrong, not for being unwell”. (Violette).*

#### Guilt

Along with fear, shame and loneliness, guilt was found to be central in the participants’ experience of maternal exhaustion. The participants tended to feel guilty even before being exhausted, at the time when they were still over-investing their maternal role. Indeed at the time, the least error would equal on their minds a failure or a fault: *“Well, I happened to think at the time that I needed some time for me. But this would make me feel guilty... Feeling so much guilty is horrible. But the worst of all is that it is not other people that make you feel guilty… you are the one to create your own guilt. And all this is because of the ideal mother I had wanted to be”. (Violette).* When finally overwhelmed, from being uncertain to be good enough mothers, the participants became certain to being inadequate mothers: *“I feel very distressed when I realize that I do not handle things properly. [...] It’s so hard, I feel so guilty when I start shouting and losing my temper. It is so much contrary to what I think an ideal parent should be.” (Elisabeth).* As pointed out earlier, at this stage, the mothers did not perceive their children as a source of joy and motivation anymore. They started to force themselves instead, which was again a source of anxiety: *“I felt so guilty to not to play with my children. Because it is widely accepted in our society that a mother should not leave her child sitting in front of the television.” (Bénédicte).* From being circumscribed and specifically related to one particular situation, the feeling of guilt evolved to becoming more general, unrelated to a specific situation, and all pervasive. The exhausted mothers saw themselves as being harmful to their children, which put their feeling of guilt at its highest: *“To know that as a mother, I put my children in danger, even if it is not deliberate, just by not looking properly after them… that’s very difficult to cope with.” (Violette).*

## Discussion

As reported in our study by five mothers who had experienced it, the experience of parental exhaustion is complex and painful. It was found to be rooted in a tendency to over-invest one’s parental role, with a desire to be perfect and an overwhelming sense of responsibility for one’s children’s future, which would leave no respite. From being happily and deliberately overinvolved, the mothers in our sample gradually developed a sense of being overwhelmed by the pressure that they perceived was being put on them. This in turn led to both physical and emotional exhaustion. At this stage, the mothers lacked the necessary resources to cope with the stress of parenthood. They reported feeling drained out and empty. Maintaining their role and responsibilities toward their normal house chores and their children became impossible. Not only did they emotionally distance themselves from their children, they sometimes lost control – verbally or physically – with them, which added even more to their distress. A sense of guilt, shame, and loneliness were central to their experience of exhaustion, as was the fear they had to harm their children and to be judged.

As our results have shown, the fear of not being a good enough mother is central to the experience of maternal burn-out. The concept of *good enough parenting* was first developed by Winnicott (1964/1991) to acknowledge that the perfect mother does not exist, and that excessive norms and undue pressure would undermine some mothers’ capacity and self-confidence. Winnicott thus acknowledged that every family is unique and that most mothers, while doing their best but not everything for their children, do a *good enough* job in taking care of their children, provided they are able to relate positively to their children, i.e., to enjoy the time spent together with them. By contrast, the mothers in our study were more concerned with aspects of doing something for rather than being with their children, with a focus on preparing for their future as adults, which would make them lose track of the quality of the present moment.

The mothers in our sample were well aware of the concept of positive parenting, which they spontaneously referred to when describing how they would set high standards and expectations regarding their role as a mother. Although the concept of positive parenting is well in line with current values and the best understanding of children’s developmental needs, our results suggest that in mothers who are prone to self-doubt and anxiety, a narrow understanding of the concept might buffer some of the mechanisms involved in parental exhaustion, such as a desire for perfection. The concept of *good enough mother*, in this context, seems interesting to recall, in order to help mothers lower their ideal goals (Hoghughi and Speight, 1998). Knowing that being perfectionist is associated with an increased vulnerability to stress (Hewitt et al., 2017), we understand how this personality trait may play a role in the occurrence of parental burnout.

Our results confirm that there are common features across the experiences of parental burnout and job burnout, such as a state of extreme fatigue, an emotional distancing from self and others, and a sense of incompetence. However, our participants’ accounts also revealed some unique aspects, in particular the sense of being trapped into an uncomfortable situation, with no way out. Whereas in situations of job burnout, people may be put on sick leave or quit their job, there is no such way out in situations of parental burnout. The responsibility of being a mother remains present no matter the level of stress and exhaustion that is experienced. The bond that mothers have to their children, although harmed and extremely fragile in practice, yet remains symbolically. Thus, even if exhausted mothers feel urged to keep their children emotionally and physically at distance, this is sometimes simply to safeguard their children from further harm they feel they might do to them if they don’t take enough distance. Moreover, it was striking from the mothers’ account how lonely and solely responsible they felt for the difficulties encountered. Unlike in situations of burnout in families with sick children, none of the mothers we have encountered has complained about objective features of their children or their family situation that would be external factors through which they would explain why they broke down. Instead, they reported developing attitudes of self-blame while pointing to their own personality and identity as a mother to explain the painful situation in which they described themselves both as victims and perpetrators. Interestingly, none of the women encountered in our study reported to regret motherhood. Instead, motherhood remained a central feature in their valued self-definition, even as they got exhausted, with the consequence that they would struggle to enhance their performance as mothers rather than wishing to go back to former social roles, when they had not yet become a mother. However, to consciously develop regret toward motherhood, i.e., the wish to “undo maternal experience”, as Donath (2015) puts it, requires considerable emotional and self-reflective work. As such, it has not been explicitly explored during the research process, and none of the mothers came up with the suggestion that they might experience regret to be mothers.

Biographic disruptions and reconstructions occur over the life span with every meaningful and stressful life event, be it positive or negative, which requires and adaptive process and challenges the sense of self. Parenthood is particularly challenging to that respect and multiple transitions are experienced as the children grow up and change. Self and identity are complex issues, which have been defined in different ways by different authors. A person’s self-concept is composed of many hierarchically organized identities according to their level of salience in relation to different social roles (Thoits, 1991). According to Sedikides and Brewer (2001), people engage in processes of self-definition by building up on 3 interrelated and co-existing self-representations, i.e., the individual self, the relational self, and the collective self. Our results suggest that exhausted mothers are challenged in all these three dimensions of self. While their *individual self* is challenged by their growing sense of incompetence and fear, their *relational self* is suffering from what we would like to call the “missed encounter with their children”, which in turn reinforces their sense of personal distress, and therefore has an impact on their individual self. As far as their *collective self* is concerned, here again it seems obvious from the participants’ accounts that some opportunities for personal empowerment through meaningful social relationships are lost, as the exhausted mothers, trapped in by their own guilt and shame, fail to ask for help. Thus, Sedikides and Brewer’s framework sheds some light on the complex nature of the self, which is multidimensional in nature. As a result of parental burnout, it seems that all these dimensions are challenged, leading to a sense of loss of self, which has been painfully expressed by the participants in our study.

Following definitions from theories of job burnout, parental burnout may be understood as occurring when resources are lacking and demands exceeding. Yet, it might be that resources are present but the mothers fail to perceive them or are reluctant to ask for help. On the one hand, mothers in such situations may be surrounded by relatives who are unaware of their distress or who feel helpless. On the other hand, they might also have inner resources which they fail to access because of a lack of self-esteem, too much fatigue or because they are not sufficiently equipped emotionally. It is likely that to identify, understand, use, express, and manage their emotions is not easy for them. Knowing the importance of emotional skills for a person’s overall wellbeing, interventions that aim to foster emotional intelligence in exhausted mothers are a promising avenue. As our interviews have shown, encouraging them to express their emotions and having creative strategies to deal with their stress and the various emotions that go through them are a good way to help them regain self-confidence and a sense of personal accomplishment related to their role as a mother.

Last but not least, the mothers’ expressed fear that they might harm their children should be taken very seriously. Although little is known about the outcomes of parental burnout on the physical, mental, and social health of the concerned children, there is sufficient evidence that children of stressed parents are at increased risk of neglect and abuse (Whipple and Webster-Stratton, 1991; Petfield et al., 2015). This risk calls for primary prevention interventions, to avoid that the children of exhausted mothers are neglected or abused.

### Strengths and Limits

To the best of our knowledge, this is the first study to give voice to very vulnerable mothers who have experienced parental burnout. We opted for a qualitative inductive method, meaning that we bracketed all *a priori* knowledge in the phase of data collection and data analysis so as to encourage the participants to voice their own concerns and their unique needs, in a non- judgmental way. Moreover, the two-step interview process allowed for a more in-depth understanding and co-interpretation with the participants themselves of their own accounts. These features make our results trustworthy and credible, according to standards in qualitative methodology (Mays and Pope, 2000). Although these results are not generalizable given the small number of interviewees, they are probably transferable to other situations, and may relevantly be applied to help design interventions to prevent or repair situations of parental burnout. Applicability of findings to other similar situations is precisely what IPA aims for, rather than generalizability (Holt and Slade, 2003).

As is typical in qualitative research, our understanding of the phenomenon under study has generated new questions that deserve further attention. First, it seems from the accounts of our participants, that the situation of parental burnout is not that of a sudden collapse which would occur overnight. On the contrary, before reaching a stage of extreme exhaustion, the participants in our study recalled how they had tried to resist and hold on, again and again. The process therefore seems progressive, with a need to keep going, as it is more difficult to quit one’s family than one’s job. The commonalities associated with different stages in this process deserve further investigation.

Another question which remains unanswered after our study is that of the role of a particular child within the family. The participants in our study have not said much about their children and the family dynamics they are part of. Yet, some of the accounts seem to suggest that in some situations one particular child in a family could be perceived as more stressful and therefore more directly associated with the maternal breakdown. As suggested by the mothers themselves, some explanations for this might be sought in their own family history and more particularly their relationship with their own mother. This calls for individualized care, to help the mothers make sense of their own history.

Last but not least, due to the characteristics of our research design, the perspective of fathers on the burnout of their partners or their own exhaustion, could not be sought. Their opinion obviously deserves to be unfolded in a separate study.

## Ethics Statement

The study was carried out in accordance with the recommendations of APA Ethical Principles of Psychologists and Code of Conduct. Written informed consent was obtained from every participant before each of the two interviews, in accordance with the Declaration of Helsinki. The protocol was approved as part of a broader research project by the Psychological Sciences Research Institute of the Université Catholique de Louvain.

## Author Contributions

SH designed the study, conducted the interviews, piloted the analysis, and drafted the manuscript. IA supervised the methodology, discussed preliminary results with SH, commented on the first draft of the manuscript, and drafted the discussion of the article. Both SH and IA approved the final manuscript.

## Conflict of Interest Statement

The authors declare that the research was conducted in the absence of any commercial or financial relationships that could be construed as a potential conflict of interest.

## Footnotes

1 http://epuisement-maternel.com

2 https://www.facebook.com/epuisementmaternel
